# Sense of coherence as moderator of the predictive power of personality variables on sexual satisfaction—a structural equation modeling approach

**DOI:** 10.3389/fpsyg.2026.1673425

**Published:** 2026-02-05

**Authors:** Aline von Hinrichs, Markus Antonius Wirtz

**Affiliations:** Public Health and Health Research, University of Education Freiburg, Freiburg, Germany

**Keywords:** personality traits, sense of coherence, sexual health, sexual satisfaction, structural equation modeling

## Abstract

**Introduction:**

The prediction of general health outcomes, such as life satisfaction and psychological wellbeing, by the *Big Five* personality traits is improved by additionally considering Sense of coherence (*SOC*). This study aims to examine whether SOC mediates the association of Big Five personality traits and *Sexual satisfaction* as psychological facet of sexual health.

**Methods:**

*N* = 206 German adults (aged 18–60) answered items on socio-demography and sexual orientation/behavior, the short Big Five Inventory (BFI-K), the SOC-L9 and the self-centered subscale of the New Sexual Satisfaction Scale (NSSS-E) in an online survey. Confirmatory factor analysis (CFA) and structural equation modeling were used (maximum likelihood estimation).

**Results:**

Model modifications ensured satisfactory to good CFA model fits for the BFI-K, SOC-L9 and NSSS-E (CFI = 0.972–0.992; RMSEA = 0.024–0.072). In particular, the SOC-L9 items had to be assigned to the two newly defined sub-factors *Sense of meaningfulness and manageability* (SOC-MM) and *Sense of comprehensibility* (SOC-CP). *Sexual satisfaction* proved to be a second-order factor underlying the first-order components *Intensity*, *Emotionality* and *Orgasm*. 20% of the variance in *Sexual satisfaction* can be predicted by *SOC-MM* (β = 0.57, *p* < 0.001; CFI = 0.940; RMSEA = 0.051), in particular. The predictive power of the Big Five-facets *Neuroticism* and *Openness* is fully mediated by *SOC-MM* (Δχ^2^_*df* = 5_ = 9.035, *p* = 0.108). Living in a partnership corresponds to higher SOC-MM and *Sexual satisfaction*. Being heterosexual also corresponds to enhanced SOC.

**Discussion:**

The association of the *Big Five* personality traits and *Sexual satisfaction* can be considered as fully mediated by *SOC-MM*. The link between *SOC-MM* for satisfaction with sexual experience and behavior thus appears to be essential in order to improve the understanding and promotion of *Sexual satisfaction* and health.

## 1 Introduction

Sexual experience and behavior are considered essential components of human health within the holistic biopsychosocial model of health (Naidoo and Wills, 2010). Accordingly, sexual health is a state of physical (e.g., reproductive or genital health), emotional (e.g., attachment, pleasure), mental (e.g., exploration, self-assertion), and social (e.g., interpersonal intimacy, interaction and communication) sexual wellbeing (World Health Organization [WHO], 2006; Lichtenberg, 2007). Similarly, *Sexual satisfaction* is defined as a multidimensional construct formed by individual’s appraisal of positive physiological, psychological, and emotional aspects of sexual experience (Anderson, 2013). It reflects the individual’s sexual affective experience and, thus, the subjective quality of one’s own sexuality (esp. sexual fulfillment; Lawrance and Byers, 1995). *Sexual satisfaction* thus constitutes a core element of subjective general human health and wellbeing (Anderson, 2013; Sánchez-Fuentes et al., 2014; Štulhofer et al., 2010).

The *New Sexual Satisfaction Scale* (*NSSS*; Štulhofer et al., 2010) was developed to assess *Sexual satisfaction*. The scale is grounded in a subjective evaluative framework, defining *Sexual satisfaction* as individuals’ personal appraisal of their sexual experiences rather than adherence to normative or performance-based standards. The NSSS is theoretically designed to be gender- and sexual-orientation inclusive, avoiding heteronormative assumptions embedded in earlier measures (e.g., Index of Sexual Satisfaction; Hudson, 1992). Overall, it advances the field by framing *Sexual satisfaction* as a positive, dynamic, and context-sensitive dimension of sexual wellbeing, rather than as the absence of sexual dysfunction. The NSSS distinguishes aspects relating to the individual’s own experience (self-centered *Sexual satisfaction*) as well as aspects relating to interactional and relational context (partner-centered *Sexual satisfaction*). Individual characteristics such as sexual mindful presence and the ability to focus on both stimuli and feelings (e.g. focus during sexual activity, NSSS-4; emotional opening up during sex; NSSS-7; see Supplementary material for complete item contents) and to experience qualitative sexual sensations as well as to perform sexually (e.g. intensity of sexual arousal; NSSS-1; sexual body response; NSSS-6) are assumed to indicate self-centered *Sexual satisfaction*. Partners’ sexual arousal and emotional openness reflect partner-centered aspects. Using the short version of the NSSS revealed that higher *Sexual satisfaction* is associated with higher education, heterosexuality, being in a relationship, partner communication about sex, relationship intimacy and satisfaction, secure attachments and coitus frequency (Hoy et al., 2018; Štulhofer et al., 2010; Khoury and Findlay, 2014). After controlling for frequency of sexual activity, older and younger people are similarly satisfied with their sexual experience (Dekker et al., 2020).

### 1.1 BIG-5 personality traits and Sense of coherence as determinants of Sexual satisfaction

Empirical studies revealed various indications that *Sexual Satisfaction* is associated with personality traits. Sexually satisfied people were found to be less neurotic, more conscientious, and open to new experiences (Jirjahn and Ottenbacher, 2023; Meltzer and McNulty, 2016). Jirjahn and Ottenbacher (2023) assume that personality shapes sexual communication and behavior in sexual relationships and, consequently, the sexual wellbeing of the individual and their partner. It is argued that the personality trait *Neuroticism*, defined as a tendency toward anxiety, nervousness, and worry, is a risk factor for sexual dissatisfaction, not only directly but also indirectly mediated through mental health. *Neuroticism* is thought to negatively affect self-confidence and mood, fears of pregnancy and of sexually transmitted diseases, feelings of security, and communication within sexual relationships. These characteristics are assumed to mediate negative effects on *Sexual satisfaction* (Strauß et al., 2012). *Conscientiousness* (i.e., the extent to which a person tends to be orderly, dutiful and responsible) and *Openness* (i.e., the extent to which a person tends to be imaginative, flexible and eager for new experiences) contribute to mutually compatible and beneficial interaction between partners, thereby strengthening the commitment of partners (Jirjahn and Ottenbacher, 2023). *Agreeableness* (i.e., the extent to which a person tends toward harmony, cooperativeness and tendered mindedness) and *Extraversion* (i.e., the extent to which a person tends to be assertive and actively sociable) proved to be of only marginal significance (r < .1) and not incrementally predictive of *Sexual Satisfacti*on.

Antonovsky (1979) emphasized the crucial importance of the *Sense of coherence (SOC)* for general health and health behavior (Eriksson and Lindström, 2006; da-Silva-Domingues et al., 2022). People are assumed to be “coherent” if they perceive their lives as comprehensible (i.e., life events are understood as structured and predictable), manageable (i.e., resources are perceived as available to meet demands) and meaningful (i.e., life challenges are considered as worthy of investment and engagement). *SOC* is regarded as essential for constructive coping in challenging situations and dealing with stressful experiences or negative expectations, as it enables and supports utilizing internal mental resources (Eriksson and Lindström, 2006; Faltermaier, 2023; Franke, 2011). In terms of *SOC* sexual health results when people experience their sexual behavior and experiences as confident, self-determined, and self-effective as well as emotionally fulfilled in accordance with their needs. In case of distressing feelings relating to one’s own sexuality (e. g. fear of failure and inadequacy, shame, lack of excitement) in challenging situations, *SOC* corresponds with higher internal and external resources that can be deployed in a protective and constructive manner. Hence, decisions relating to sexual behavior are made in a more responsible, thoughtful, and self-confident and emancipated manner (Stumpe, 2012).

Furthermore, SOC components may increase sexual satisfaction, as individuals who report, for example, a higher sense of meaningfulness may also experience greater perceived emotional connectedness and partner intimacy, which in turn are associated with sexual satisfaction (Anderson, 2013). Consequently, fulfillment of sexual desire, experience of sexual pleasure, physical health, and psychological wellbeing may be promoted by *SOC*—and vice versa. Accordingly, Stumpe (2012) claims *SOC* to be a decisive predisposition for healthy sexuality and sexual health. Furthermore, Lashani et al. (2021) found that *SOC* fully mediates the relationship between general health and *sexual functioning* and *satisfaction*, respectively. Armeni et al. (2023) were able to verify these findings and demonstrated that the significant association between *SOC* and sexual functioning and satisfaction is not affected by age, depressive symptoms, diabetes, hypertension and the influence of menopausal hormone therapy.

Barańcuk (2021) examined the three construct domains *Big Five, SOC* and *General health* in a meta-analysis. She confirms that *Big Five* personality traits and *SOC* are important determinants of mental, physical, and social health. Particularly, higher *Neuroticism* and lower *SOC* are considered to result in poorer mental and physical health. This may be attributed to the fact that *Neuroticism* and *Coherence* both affect peoples’ health-conscious behaviors, which in turn can activate the body’s own resources and strengthen resilience.

*Sexual satisfaction* is not only determined by individual factors (e.g., personality traits, emotional intelligence or self-esteem, social skills and social support, sexual functioning; Ambrosini and Biolcati, 2025; Henderson et al., 2008; Weber et al., 2024), attitudes (e.g., attachment avoidance/-anxiety, positive and respectful approach, sexual self-comfort; Ambrosini and Biolcati, 2025; World Health Organization [WHO], 2006; Higgins et al., 2011) and behaviors (e.g., sensation seeking; Pechorro et al., 2016), but also by socially shaped conditions and opportunities to gain safe and self-determined sexual experiences (Channon et al., 2010; Higgins et al., 2022). Particularly for the LGBTIQ+ community, the significance of *Sexual minority stress* caused by social prejudice against sexual minorities through stigmatization is assumed to be influential (Frost et al., 2015). One central proximal mechanism is considered to be internalized *homonegativity*, which reflects the interiorization of negative social attitudes toward one’s own sexual orientation (Cienfuegos-Szalay et al., 2022). Internalized *homonegativity* primarily manifests itself through sexual shame, self-conscious feelings associated with self-devaluation of one’s own sexuality. Sexual shame affects sexual self-acceptance and intimacy, thereby reducing the ability to have pleasurable and affirming sexual experiences. Anderson (2013) emphasizes the bidirectional, reciprocal importance of *Sexual satisfaction* and mental and physical health.

### 1.2 Aims of this study

The *Big Five personality traits* are assumed to be determinants of both *SOC* (Grevenstein and Bluemke, 2015; Barańcuk, 2021) and health aspects, like sexual health (Jirjahn and Ottenbacher, 2023). Furthermore, the *SOC* determines general and sexual health (da-Silva-Domingues et al., 2022; Lashani et al., 2021). In this study, the relationship between the *Big Five, SOC* and *Sexual satisfaction*, as a proxy of sexual health, will be examined for the first time in a joint structural path model. The aim is to examine whether the predictive power of the *Big Five* for *Sexual Satisfaction* can be assumed to be mediated by *SOC*. The hypotheses are as follows:

*H1*: The Big Five personality traits *Extraversion*, *Agreeableness, Conscientiousness*, and *Openness* are positive predictors of *Sexual satisfaction. Neuroticism* is a negative predictor of *Sexual satisfaction*.

*H2*: *SOC* is a positive predictor of *Sexual satisfaction*.

*H3*: The predictive power of the Big Five personality traits *Extraversion*, *Agreeableness*, *Conscientiousness*, *Neuroticism*, and *Openness* for *Sexual satisfaction* is fully mediated by *SOC*.

*H4*: Gender, age, partner status and sexual orientation are correlated with the *Big Five constructs*, *SOC* and *Sexual satisfaction*.

## 2 Materials and methods

### 2.1 Data collection and study sample

Data collection was conducted using an online questionnaire from October to December 2024. The questionnaire was pre-tested by N = 8 adult person using comprehension probing (Pohontsch and Meyer, 2015). Adults aged between 18 and 60 were eligible to participate in the survey. The study was primarily advertised via flyers in southwestern Germany and via email distribution lists and social networks (snowball system). Each participant was rewarded with a donation of €1-2 to a charitable organization. Informed consent was obtained from all participants. The study has been approved by the Ethics Committee of the University of Education in Freiburg (PHFR 25-12). The average processing time for the questionnaire was M = 16.5 min (SD = 4.7, MIN = 5.5; MAX = 30.9).

### 2.2 Assessment instruments

Socio-demographic data collected included non-binary and trans*-inclusive gender (Diethold, 2020), age, sexual orientation and subjectively perceived socio-economic (MacArthur Scale; Hoebel et al., 2015). In addition, the frequency of sexual activity in the last 4 weeks and the number of sexual partners in the last 12 months were surveyed using validated single items (Dekker et al., 2020).

The Big Five personality traits were assessed using the 21 five-point Likert-scaled items (“strongly disagree” to “strongly agree”) of the German short version of the Big Five Inventory (Rammstedt and John, 2005). Internal consistency proved to be acceptable to good for Extraversion (α = 0.81/.86), Neuroticism = 0.74/.77, and *Conscientiousness* = 0.69/.70. The values for Agreeableness (0.59/0.64) and Openness (0.66/0.70) fell below the thresholds for acceptable fit. Arens and Preckel (2025) report, that the 5-dimensional structure of the BFI-K could only be confirmed after considering residual correlations, which correct polarization effects in particular.

The German Leipzig short version SOC-L9 (Schumacher et al., 2000) assesses SOC. Four items are answered on a bipolar seven-point response scale (“1 = very often” to “7 = very rarely or never”). For the remaining five items, sentences must be completed (e.g., “When you think about your life, is it very often the case that…” with the poles “You wonder why you are alive at all” and “You feel how good it is to be alive”). As the 3-factorial structure of the SOC (29 items) and the SOC-13 (13 items) could not be sufficiently substantiated, the SOC-L9 was developed as a one-dimensional scale (Cronbach’s α = 0.87).

Self-centered *Sexual satisfaction* was assessed using the 10 items of the corresponding subscale of the New Sexual Satisfaction Scale (NSSS) (Brouillard et al., 2020), which proved to be highly internally consistent with α ≥ 0.88 (Pechorro et al., 2016, Štulhofer et al., 2010). Six of the 10 items have already been translated and validated in German language (NSSS-D) (Hoy et al., 2018). The four remaining original NSSS items were translated in collaboration with a qualified translator with extensive professional experience. In accordance with the recommendation by Hoy et al. (2018), the partner- and activity-oriented NSSS subscales were not included in the survey, as the study was intended to include singles and individuals without prior interpersonal sexual experiences. Further dimensions of sexual health were surveyed (e.g., sexual attitudes, sexual pride and shame), but these are not the subject of this study.

### 2.3 Data analysis

Study participants who left no more than three items unanswered for each of the three assessment instruments were removed from the data set due to insufficient data information. In the case of individual missing responses, a maximum likelihood-based imputation was carried out using the Expectation Maximization algorithm (Schafer and Graham, 2002). This is recommended in order to minimize potential biases in case of not-completely-random missing values (Wirtz, 2004). MacDonald’s ω was calculated as measure of internal consistency (Hayes and Coutts, 2020).

A three-step procedure was applied to test the study hypotheses. (1) The latent structure of the individual instruments was tested by means of confirmatory factor analysis (CFA) using maximum likelihood estimation (Kline, 2016). The data compatibility of the model was assessed using measures of global and local fit (Kline, 2016). Non-significant values [*p*(χ^2^) > 0.05) indicate that there are no systematic differences between model predictions and data structures at the variance-covariance matrix level. Due to its overly high sensitivity to marginal model deficiencies, the Confirmatory Fit Index (CFI) and the Tucker-Lewis Index (TLI) as well as the Root Mean Square Error of Approximation (RMSEA) provide more appropriate assessments of model fit (acceptable/good: CFI/TLI: > 0.95/ > 0.97; RMSEA < 0.08/0.05; Schermelleh-Engel et al., 2003). Kline (2016) recommends lower critical values for incremental fit measures: CFI, TLI > 0.90: acceptable; > 0.95 good. At the local level, factor loadings > 0.63 indicate a sufficient association of the according item and the underlying construct (Kline, 2016). (2) Subsequently, the predictive structures between the underlying constructs are modeled integratively in a structural equation model (SEM). According to the hypotheses, (i) the *Big Five* constructs are defined as predictors of SOC and *Sexual satisfaction* and (ii) SOC as a predictor of *Sexual satisfaction*. (3) In a hierarchical model comparison, it is examined whether the direct paths of the *Big Five* are required as predictors of *Sexual satisfaction* or whether these can be assumed to be fully mediated by SOC. If there is no significant decrease in data fit after fixing the direct paths to 0 [*p*(Δχ^2^, df = 5) > 0.05; nested model comparison; Kline, 2016), a complete mediation can be assumed. The model estimates are determined using the AMOS 29.0 maximum likelihood estimation software (Arbuckle, 2022).

## 3 Results

### 3.1 Sample characteristics

After excluding *N* = 5 persons with an unacceptable proportion of missing data (at least *n* = 13 missing data on the 40 analysis items), *N* = 206 adults formed the final analysis sample (Table 1). The participants were mostly female (*n* = 141; 68.4%) and heterosexual (*n* = 129; 62.6%). Note, that a high proportion of participants *n* = 44 (21.4%) reported being bisexual. In a German national representative survey, the corresponding proportion was only 1.3% (Dekker et al., 2020). Moreover, the sample is rather young (*M* = 28.4 years; median: 24.0 years), with higher socioeconomic status: *M* = 6.32 (SD = 1.46; Germany representative comparative data: *M* = 5.3/5.2, SD = 1.6/1.7; Hoebel et al., 2015). *n* = 90 (43.7%) live in a steady relationship, *N* = 80 (38.8%) are single. People living in a steady relationship were sexually active in the last 4 weeks most frequently (*M* = 6.3; median 4; *n* = 85 valid responses), and singles least frequently (*M* = 1.3; Md = 0; *n* = 67 valid responses). Regarding national reference data, women with *M* = 4.1 in the present sample have sexual intercourse more frequently and men with *M* = 3.7 somewhat less frequently than the average of the German population (Hoy et al., 2018: M(women) = 3.3; M(men) = 4.2). *n* = 118 (59.6% *N* = 198 valid responses) reported having one partner in the last year, *N* = 22 people (11.1%) stated that they had no partner. *M* = 3.74 (SD = 0.80) indicated that *Sexual satisfaction* on the NSSS-E was slightly lower than in the nationally representative survey in Germany by Hoy et al. (2018) with *M* = 3.96 (SD = 0.78). However, it should be noted that the mean value reported by Hoy et al. (2018) is elevated because data from sexually inactive individuals were not included.

**TABLE 1:** Sample characteristics (*N* = 206).

Characteristic	Categories		Number	Percentage	
Gender	Female		141	68.8%	
	Male		58	28.3%	
	Non-binary^a^		6	2.9%	
Sexual orientation	Heterosexual		129	62.6%	
	Homosexual		11	5.3%	
	Bisexual		44	21.4%	
	Pansexual		16	7.8%	
	Asexual		4	1.9%	
	“Not listed”		2	1.0%	
Relationship status	Single		80	38.8%	
	Committed relationship		90	43.7%	
	Married		27	13.1%	
	“It’s complicated”		9	4.4%	
Sexual activity in the last 4 weeks (valid responses: *n* > 182)	0		54	29.7%^a^	
	1–4		72	39.5%	
	5–9		30	16.5%	
	10–14		14	7.7%	
	15–19		6	3.3%	
	>19		5	3.2%	
Sexual partners in the last 12 months (valid responses: *n* > 198)	0		22	11.1%^a^	
	1		118	59.6%	
	2		20	10.1%	
	3		10	5.1%	
	4		15	7.6%	
	5		5	2.5%	
	>5		8	4%	
	**M**	**SD**	**Median**	**MIN**	**MAX**
Age	28.36	9.99	24.00	18	60
Subjective socioeconomic status^b^	6.31	1.46	7	2	9

^a^Valid percentage.

^b^Mac Arthur scale.

### 3.2 Confirmatory factor analysis of the BFI-K, SOC-L9 and NSSS- self-centered scale

As reported by Arens and Preckel (2025) for secondary school students, the five-dimensional model of the BFI-K does not fit adequately in the present sample either (CFI = 0.822; TLI = 0.791; RMSEA = 0.079; Table 2). Due to insufficient loadings (< 0.63), two items were eliminated for each of the factors *Agreeableness* and *Conscientiousness*, one item for each of the factors *Extraversion* and *Neuroticism*, and three items for *Openness*. The resulting model with 12 items showed an acceptable fit (CFI = 0.944; TLI = 0.923; RMSEA = 0.063) with weakly to moderately correlated factors (max | r| = 0.30; *Conscientiousness* and *Neuroticism*). Table 3 shows the descriptive BFI-K item and scale characteristics.

**TABLE 2:** CFA global fit measures for the BFI-K, the SOC-L9 and the NSSS self-/acitivity-focused subscale.

Models	Items	χ ^2^	df	p	χ ^2^/df	CFI	TLI	RMSEA
Acceptable fit				>0.05	<3	>0.95	>0.95	<0.08
Good fit					<2	>0.97	>0.97	<0.05
**BFI-K**								
Original model	21	406.42	179	<0.001	2.27	0.822	0.791	0.079 (0.069; 0.089)
Modified model	12	103.53	57	<0.001	1.82	0.944	0.923	0.063 (0.043; 0.082)
**SOC-L9**								
Original model	9	65.40	27	<0.001	2.42	0.931	0.908	0.083 (0.058; 0.109)
Modified model	9	26.80	24	0.314	1.12	0.995	0.992	0.024 (0.000; 0.063)
**NSSS subscale**								
Original model	10	234.93	35	< 0.001	6.71	0.820	0.769	0.167 (0.147; 0.187)
Modified model	9	51.77	25	0.001	2.07	0.972	0.960	0.072 (0.044; 0.100)

**TABLE 3:** Descriptive item and scale characteristics of the BFI-K.

I am someone who	M	SD	r_it,c_^a^	I.R.^b^	C.R.^c^
**Extraversion (ω = 0.810)**					
E01: Is more shy ^R^	3.82	0.95	0.398	0.59	–^d^
E03: Tends to be the quiet type ^R^	3.48	0.92	0.643	0.66	9.29
E04: Is outgoing, sociable	3.13	0.95	0.629	0.49	8.93
**Deleted:**					
E02: Is enthusiastic and can easily inspire others.	3.45	1.11	0.651	–	–
**Agreeableness (α = 0.743)**					
A03: Can behave in a cold and distant manner ^R^	3.14	1.13	0.561	0.54	–^d^
A04: Can behave harshly/dismissively toward others ^R^	3.41	1.02	0.604	0.64	–^d^
**Deleted**					
A01: Tends to criticize others ^R^	3.34	0.94	0.394	–	–
A02: Easily trust others, believes in the good in people	3.79	1.00	0.387	–	–
**EConscientiousness (α = 0.659)**					
C02: Is comfortable, tends to be lazy ^R^	2.98	1.11	0.587	0.40	–^d^
C04: Makes plans and execute them	3.66	0.87	0.546	0.61	–^d^
**Deleted**					
C01: Completes tasks thoroughly	3.87	0.85	0.455	–	–
C03: Is efficient and works quickly	3.55	0.96	0.583	–	–
**Openness (α = 0.856)**					
O04: Appreciates art and aesthetic impressions	4.06	1.06	0.626	0.86	–^d^
O05: Has little interest in art ^R^	3.82	1.19	0.636	0.66	–^d^
**Deleted**					
O01: Has a wide range of interests	4.18	0.89	0.330	–	–
O02: Is profound, likes to think about things	4.17	0.83	0.315	–	–
O03: Has an active sense of imagination	3.95	1.05	0.466	–	–
**Neuroticism (ω = 0.790)**					
N01: Easily becomes downcast or depressed	3.07	1.12	0.617	0.46	–^d^
N03: Worries a lot	3.48	1.11	0.685	0.69	8.34
N04: Gets nervous and insecure easily	3.07	1.09	0.611	0.53	8.29
**Deleted**					
N02: Is relaxed, does not let stress upset me ^R^	3.13	1.13	0.530	–	–

Response format: “1,” “strongly disagree”; “5,” strongly agree”; ^R^, Reverse item polarity.

^a^Corrected item-total-correlation.

^b^Indicator reliability.

^c^Critical ratio of unstandardized item loadings, *p* < 0.001 for all items.

^d^Unstandardized loading restricted to 1 to allow for identification.

The original unidimensional SOC L9 model revealed a global model fit of CFI = 0.931, TLI = 0.908, and RMSEA = 0.083 (Table 2). On local fit level, items SOC-L1 and SOC-L4 exhibited insufficient loadings. In addition, their residual correlation was significantly positive at *r* = 0.29. Since these items are also theoretically assigned to the *Comprehensibility* aspect, a corresponding subconstruct was defined in a modified two-factor model. After the significant residual correlations of items SOC-L3 and SOC-L9 (*r* = 0.28) as well as SOC-L6 and SOC-L7 (*r* = –0.29) were included in this modified model, a very good global fit was achieved: CFI = 0.995, TLI = 0.992, RMSEA = 0.024. Table 4 shows the descriptive SOC-L9 item and scale characteristics.

**TABLE 4:** Descriptive item and scale characteristics of the SOC-L9.

Scales/items	M	SD	r_it,c_^a^	I.R.^b^	C.R.^c^
**SOC-MM (manageability/meaningfulness) (ω = 0.825)**					
SOC-MM1: It’s good to be alive	5.12	1.53	0.688	0.61	–^d^
SOC-MM2: Everyday things are a source of pleasure	5.01	1.27	0.589	0.38	8.53
SOC-MM3: Feeling good is persistent	5.18	1.47	0.525	0.34	8.04
SOC-MM4: Future life is full of sense and purpose	5.62	1.30	0.563	0.44	9.00
SOC-MM5: Not feeling like a looser	3.95	1.76	0.521	0.41	8.63
SOC-MM6: Feeling of overcoming difficutlies	4.99	1.42	0.468	0.27	7.17
SOC-MM7: Never feeling things are meaningless	4.61	1.59	0.626	0.42	9.08
**SOC-CP (comprehensibility) (α = 0.650)**					
SOC-CP1: Not feeling insecure in unfamiliar situations	4.52	1.51	0.693	0.48	–^d^
SOC-CP2: Never having mixed-up feelings and ideas	3.79	1.62	0.693	0.48	–^d^

Response format: “1,” “strongly disagree”; “5,” “strongly agree.”

^a^Corrected item-total-correlation.

^b^Indicator reliability.

^c^Critical ratio of unstandardized item loadings, *p* < 0.001 for all items.

^d^Unstandardized loading restricted to 1 to allow for identification

CFI = 0.820, TLI = 0.769, and RMSEA = 0.167 indicated distinct violations of the unidimensionality of the 10 NSSS self-centered items (Table 2). Residual correlations suggested three sub-facets *Intensity* (5 items; Table 5), *Emotion* (2 items) and *Orgasm* (2 items). The item NSSS-6 (sexual body reactions) had to be excluded from the model as it did not show a substantial loading on any of the three subfactors. The 2nd order factor *Sexual satisfaction* explains 82% of the variance of *Intensity* and *Emotion* as well as 44% of the variance of *Orgasm*. The 2nd-order model showed an acceptable to good global fit: *CFI* = 0.972, *TLI* = 0.960, *RMSEA* = .0072.

**TABLE 5:** Descriptive item and scale characteristics of the NSSS self-/activity-focused subscale (McDonald’s ω of total 9-item-scale: 0.893).

How satisfied are you with	M	SD	r_it,c_^a^	I.R.^b^	C.R.^c^
**Intensity (ω = 0.861)**					
Int01: Intensity of sexual arousal	3.71	1.05	0.637	0.49	–^d^
Int02: Letting go and surrender to sexual pleasure	3.80	1.00	0.722	0.71	10.83
Int03: Focus/concentration during sexual activity	3.62	1.04	0.689	0.59	10.02
Int04: Sexual reacting to partner	3.87	1.03	0.703	0.58	9.88
Int05: Providing pleasure to partner	3.93	1.03	0.632	0.42	8.51
**Emotionality/Reciprocity (α = 0.796)**					
Em01: Emotional opening up in sex	3.76	1.12	0.692	0.68	–^d^
Em02: Mood after sexual actitivty	3.83	1.13	0.665	0.64	–^d^
**Orgasm (α = 0.841)**					
Og04: Quality of orgasms	3.67	1.18	0.618	0.77	–^d^
Og05: Frequency of orgasms	3.42	1.24	0.573	0.68	–^d^
**Deleted**					
Body’s sexual functioning	3.83	1.01	–	–	–

Response format: “1,” “not at all satisfied”; “5,” “extremely satisfied” (time reference: last 6 month).

^a^Corrected item-total-correlation.

^b^Indicator reliability.

^c^Critical ratio of unstandardized item loadings, *p* < 0.001 for all items.

^d^Unstandardized loading restricted to 1 to allow for identification.

### 3.3 Testing the SEM assuming BIG-5 personality traits and SOC to predict Sexual satisfaction

In an initial SEM model the BIG-5 constructs *Extraversion*, *Agreeableness*, *Conscientiousness*, *Neuroticism* and *Openness* determined 17% of the variance of *Sexual satisfaction* (CFI = 0.951, TLI = 0.942, RMSEA = 0.047; Table 6). *Neuroticism* (β = –0.321, *p* < 0.001) and *Openness* (β = 0.177, *p* = 0.025) proved to be significant predictors (Hypothesis 1 partially confirmed). The second SEM model, containing only *SOC-MM* and *SOC-CP* as predictors, explained 18% of the variance in *Sexual satisfaction* (CFI = 0.965, TLI = 0.958, RMSEA = 0.046). Only *SOC-MM* proved to be a significant predictor (β = 0.368, *p* = 0.023) (Hypothesis 2 partially confirmed).

**TABLE 6:** Measures of global fit for the structural equation model (SEM) of Big Five, SOC and NSSS.

Models	χ ^2^	df	*p*	χ ^2^/df	CFI	TLI	RMSEA
Acceptable fit			>0.05	<3	>0.950	>0.950	<0.08
Good fit			0	< 2	> 0.970	>0.970	< 0.05
Partially SOC mediated Model	644.73	407	<0.001	1.58	0,913	0.900	0.053 (0.045, 0.061)
Fully SOC mediated Model	652.53	412	<0.001	1.58	0.911	0.900	0.053 (0.046; 0.061)
Δχ^2^_df = 1_	7.80	5	0.168				

Subsequently, an integrative latent path model was defined to determine the predictive value of (i) the *Big Five* personality traits and (ii) *SOC-MM* and *SOC-CP* for *Sexual satisfaction*. First, a model was estimated, assuming a partial mediation of the predictive value of the *Big Five* personality traits via *SOC-MM* and *SOC-CP* (*partially mediated model*). Second, the model assuming no direct predictive value of the BIG-5 personality traits on *Sexual satisfaction* was estimated (*fully mediated model*). The fit of the nested, fully mediated model (CFI = 0.911, TLI = 0.900, RMSEA = 0.053; Table 6) proved to be not significantly poorer than the fit of the partially mediated model (CFI = 0.911, TLI = 0.900, RMSEA = 0.053) (Δχ^2^_*df*__=__5_ = 7.80; *p* = 0.168). Accordingly, it is suggested that the BIG-5 have no predictive value for *Sexual satisfaction* beyond *SOC-MM* (Hypothesis 3 confirmed). Note, *Agreeableness* (β = 0.274, *p* = 0.003) and *Neuroticism* (β = –0.815, *p* < 0. 001) predict *SOC-CP* to 93%: This very high predictive path suggests that *Neuroticism* and *SOC-CP* should be considered virtually identical. The variance of the construct *SOC-MM* is explained by 75%, with *Agreeableness* (β = –186. *p* = 0.021) and *Neuroticism* (β = –0.934, *p* < 0.001) proving to be significant predictors. While *Sexual satisfaction* (19% variance explained) is significantly predicted by *SOC-MM* (β = 0.443, *p* = 0.005), it is not predicted by *SOC-CP* (β = –0.016, *p* = 0.916). Note that in this structural model, the correlation between the endogenous error terms of SOC-CP and *SOC-MM* no longer exists [t_*df*__=__205_ (*r* = 0.213) = 0.341; *p* = 0.733]. This means that after controlling the common prediction components of the *Big Five SOC-CP* and *SOC-MM* share no more common variance anymore (*r* = 0.76, *p* < 0.001).

### 3.4 Mean differences in BIG-5 personality traits, SOC and Sexual satisfaction

Table 7 shows that women (*d* = 0.44) and people living in partnerships (*d* = –0.41) have higher scores for *Conscientiousness*. Women also report higher *Neuroticism* scores (*d* = 0.48). *SOC-MM* is more pronounced for people living in a relationship (*d* = –0.39) and heterosexual people (*d* = 0.45). With regard to *Sexual satisfaction*, consistently higher scores are found for people living in relationships (*d* = –0.50 to –0.27). People aged 30 and over reported increased emotional and orgasmic *Sexual satisfaction* (*d* = –0.39, –0.34) (Hypothesis 4 partially confirmed).

**TABLE 7:** Mean values on the BFI-K. SOC-L9 and NSSS scales for gender, age, partner status and sexual orientation.

*N* =	Total	Gender			Age			Partner status			Orientation		
	206	Female 141	Male 56	d (CI)^c^	<30 146	≥30 60	d (CI)^c^	Single 89	Partner 117	d (CI)^c^	H^a^ 131	O^b^ 75	d (CI)^c^
	M (SD)	M (SD)	M (SD)		M (SD)	M (SD)		M (SD)	M (SD)		M (SD)	M (SD)	
**BFI-K**													
Extra-version	3.49 (0.76)	3.57 (0.71)	3.36 (0.86)	0.28 (−0.03 to 0.59)	3.54 (0.76)	3.36 (0.74)	0.24 (−0.06 to 0.54)	3.53 (0.72)	3.46 (0.79)	0.08 (−0.20 to 0.35)	3.50 (0.78)	3.46 (0.71)	0.06 (−0.22 to 0.35)
Agreeableness	3,43 (0.74)	3.45 (0.74)	3.38 (0.77)	0.08 (−0.22 to 0.39)	3.38 (0.71)	3.55 (0.79)	−0.23 (−0.53 to 0.07)	3.30 (0.73)	3.53 (0.74)	−0.31 (−0.60 to 0.04)	3.44 (0.73)	3.42 (0.76)	0.02 (−0.27 to 0.30)
Conscientiousness	3.53 (0.72)	3.64 (0.67)	3.33 (0.77)	0.44*** (0.13 to 0.75)	3.48 (0.74)	3.63 (0.65)	−0.21 (−0.51 to 0–09)	3.37 (0.69)	3.65 (0.71)	−0.41 *** (−0.69 to 0.13)	3.56 (0.69)	3.48 (0.75)	0.11 (−0.18 to 0.39)
Openness	404 (0.69)	4.01 (0.69)	4.07 (0.72)	−0.09 (−0.40 to 0.21)	4.04 (0.71)	4.02 (0.66)	0.04 (−0.26 to 0.34)	4.08 (0.57)	4.00 (0.78)	−0.12 (−0.16 to 0.40)	3.99 (0.69)	4.12 (0.69)	−0.20 (−0.48 to 0.09)
Neuroticism	3.16 (0.87)	3.27 (0.85)	2.85 (0.90)	0.48 *** (0.17 to 0.79)	3.23 (0.84)	3.00 (0.93)	0.26 (−0.04 to 0.56)	3.13 (0.85)	3.19 (0.89)	−0.06 (−0.33 to 0.22)	3.09 (0.90)	3.30 (0.81)	−0.24 (−0.52 to 0.05)
**SOC-L9**													
SOC-MM	4.93 (1.03)	4.98 (1.06)	4.87 (0.98)	0.10 (−0.20 to 0.41)	4.85 (1.02)	5.11 (1.06)	−0.26 (−0.56 to 0.05)	4.70 (1.04)	5.10 (1.00)	−0.39*** (−0.67 to 0.12)	5.10 (0.94)	4.64 (1.13)	0.45*** (0.16 to 0.73)
SOC-CP	4.16 (1.35)	4.14 (1.39)	4.32 (1.24)	−0.13 (−0.44 to 0.18)	4.09 (1.33)	4.33 (1.39)	−0.18 (−0.49 to 0.12)	4.13 (1.31)	4.18 (1.38)	−0.04 (−0.31 to 0.24)	4.29 (1.36)	3.93 (1.30)	0.26 (−0.02 to 0.55)
**NSSS**													
Overall	3.73 (0.81)	3.68 (0.83)	3.89 (0.72)	−0.27 (−0.57 to 0.04)	3.67 (0.82)	3.88 (0.77)	−0.25 (−0.56 to 0.05)	3.55 (0.73)	3.87 (0.83)	−0.41*** (−0.69 to 0.13)	3.80 (0.73)	3.63 (0.92)	0.21 (−0.08 to 0.49)
Intensity	3.79 (0.83)	3.73 (0.85)	3.94 (0.77)	−0.26 (−0.56 to 0.05)	3.77 (0.83)	3.83 (0.84)	−0.07 (−0.38 to 0.23)	3.66 (0.76)	3.88 (0.87)	−0.27 * (−0.55 to 0.01)	3.83 (0.74)	3.71 (0.96)	0.14 (−0.15 to 0.42)
Emotion	3.79 (1.02)	3.75 (1.08)	3.92 (0.90)	−0.17 (−0.48 to 0.14)	3.68 (1.07)	4.07 (0.83)	−0.39 ** (−0.69 to 0.08)	3.51 (1.00)	4.00 (0.99)	−0.50 *** (−0.78 to 0.22)	3.90 (0.95)	3.61 (1.12)	0.28 (−0.01 to 0.56)
Orgasm	3.54 (1.12)	3.49 (1.20)	3.74 (0.83)	−0.23 (−0.54 to 0.08)	3.43 (1.14)	3.81 (1.04)	−0.34* (−0.64 to 0.03)	3.31 (1.04)	3.72 (1.15)	−0.37 ** (−0.65 to 0.10)	3.61 (1.05)	3.42 (1.24)	0.17 (−0.11 to 0.46)

*p < 0.05, **p < 0.01; ***p < 0.001 (*t*-test for independent groups); ^a^H = Heterosexual, ^b^(now associated with ‘Heterosexual’) ‘O = Other’, ^c^Cohen’s *d* and 95%-Confidence interval. Bold faced: significant values.

## 4 Discussion

This study was the first to examine the relationship between the *Big Five* personality traits and *Sexual satisfaction* with regard to the mediating role of *SOC*. The model assuming the *SOC* constructs to fully mediate the predictive value of the *Big Five* on *Sexual satisfaction* best explained the empirical data structures. The *Big Five* personality traits *Agreeableness*, *Conscientiousness* and *Neuroticism* predict *SOC-MM* (76% variance explained), and *SOC-MM* predict *Sexual satisfaction* (18% variance explained).

Our results are consistent with those found by Jirjahn and Ottenbacher (2023), where *Neuroticism* had the highest predictive value for *Sexual satisfaction*. *Neuroticism* was the only *Big Five* personality trait with medium predictive effect size. Due to the very large sample size (*N* = 6.240), the other four *Big Five* predictors proved also to be significant despite small or marginal effect sizes even below β = 0.10. The results of the present study suggest that the relationships between the *Big Five* personality traits and *Sexual satisfaction* reported by of Jirjahn and Ottenbacher (2023) are completely mediated by *SOC-MM* [esp. β(*Neuroticism* → *SOC-MM*): –0.686; β(*SOC-MM* →*Sexual satisfaction*): 0.443].

The results for hypotheses 2 and 3 support the findings of Lashani et al. (2021) and Armeni et al. (2023) in a clinical sample of women, though they, however, found only a weak to moderate predictive value of *SOC* for *Sexual functioning* and *satisfaction* (max. β = 0.150) using logistic regression. Our results show, on the one hand, that the *Big Five* personality traits have no incremental predictive value beyond *SOC-MM*, and that the associations are more pronounced in our non-clinical sample with β = 0.443.

To ensure sufficient factorial validity for the BFI-K, the SOC-L9 and the self-focused subscale of the NSSS in confirmatory modeling, adjustments had to be made. Unlike Arens and Preckel (2025), who used a bifactorial model for the BFI-K, the present study achieved an appropriate five-factor CFA structure by eliminating items with insufficient loadings. The final modified CFA model is based on 12 of the original 21 items (Table 2). However, the reliability of the subscales between 0.7 and 0.8 and clearly lower construct correlations indicate acceptable construct validity of the BFI-K despite the reduced number of items. When interpreting the results, it must be borne in mind that the corresponding constructs represent a reduced range of content. Along with the results of Arens and Preckel (2025), implications for the factorial and construct validity of the BFI-K should be examined in more detail, particularly with regard to the original version of the BFI (Rammstedt and Danner, 2017).

After defining two items as indicators of a new factor *SOC-CP*, a good model fit was also achieved for the SOC-L9. Generally, Singer and Brähler (2014) also suggested that SOC-L9 items cannot be considered strictly one-dimensional or three-dimensional, and that modifications are necessary to ensure construct validity. In line with their suggestions the two-dimensional structure found to be valid in the present study could provide the basis for a more general psychometrically convincing SOC-L9 structure. Moreover, one-dimensional measurement and modeling of SOC should be viewed critically in principle and in terms of its significance for sexuality, as different mechanisms of action are assumed for the three SOC subcomponents. Comprehensibility influences wellbeing through cognitive mechanisms, as perceiving experiences as structured and predictable reduces uncertainty and stress. Manageability operates via a resource-based mechanism, enabling active coping and a sense of control by mobilizing internal and external resources. Meaningfulness affects wellbeing through a motivational–emotional mechanism, increasing engagement, persistence, and willingness to invest effort in dealing with life’s challenges (Antonovsky, 1979; Eriksson and Lindström, 2006). E.g., the component of meaningfulness, for example, may result in (and through) higher perceived emotional connection and partnership intimacy. The findings explored in this study should contribute to a more differentiated view of SOC. In independent validation studies and other application contexts, their significance for construct understanding and moderating contextual effects should be investigated.

**FIGURE 1:**
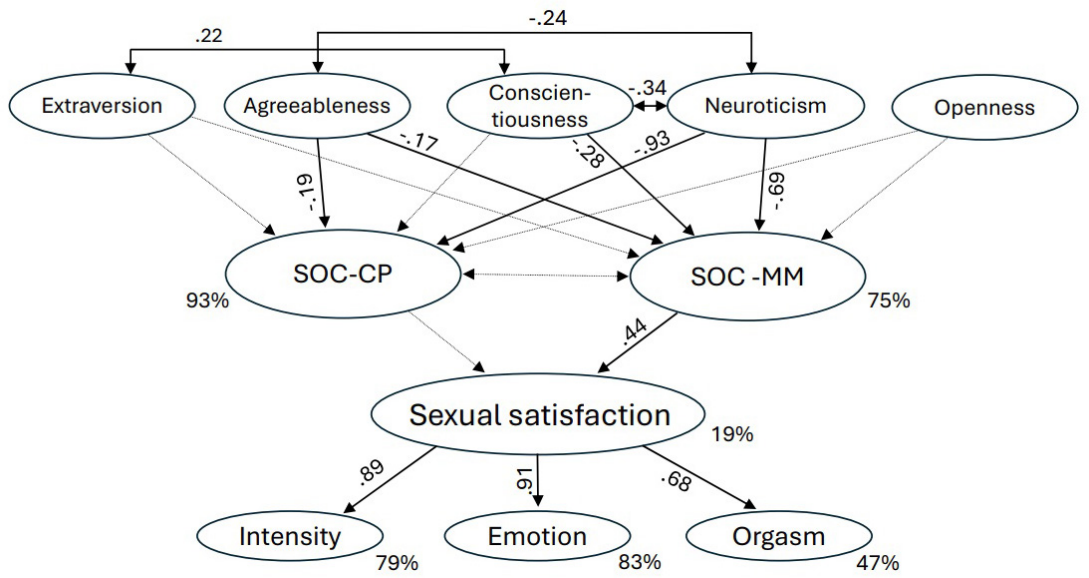
Final fully mediated structural model. Labelled, solid arrows are significant.

Although the *Big Five* constructs (esp. *Neuroticism*) and *SOC* proved to be critically highly correlated (Feldt et al., 2007; Hochwälder, 2012), Grevenstein and Bluemke (2015) have shown that *SOC* has an incremental predictive value for health status traits in addition to the *Big Five* personality traits. The separation of *SOC-CP* and *SOC-MM* mitigated this problem in the present study, enabling a more differentiated and informative modeling of the data structure. Our results revealed a strikingly high redundancy of *SOC-CP* and the BFI-K factor *Neuroticism* (β (*Neuroticism* → *SOC-CP*) = –0.93). However, *SOC-MM* is clearly better differentiated with β(*Neuroticism* → *SOC-MM*) = –0.69). Moreover, the structural model (Figure 1) no longer shows any significant residual correlations between *SOC-CP* and *SOC-MM*. This means that after controlling for the *Big Five* information components (esp. *Neuroticism*), the two sub-constructs *SOC-CP* and *SOC-MM* may therefore be regarded as even statistically independent. According to our findings, the incremental information content of *SOC* compared to *Neuroticism*, as identified by Grevenstein and Bluemke (2015), can thus be attributed primarily to the *SOC* sub-aspects of *Meaningfulness* and *Manageability*. This provides important starting points for both measuring *SOC* and modeling its associations. Instead of solely considering the aggregated *SOC* total score, it seems warranted to evaluate comparatively whether the constituent *SOC* sub-aspects of *Comprehensibility*, *Meaningfulness*, and *Manageability* result in statistically and contextually more valid component and structural modeling.

It would also be important to consider the relationship between *SOC-CP* and *SOC-MM* and *eudaemonic* and *hedonic wellbeing* (Diener et al., 2018). According to Ryff et al. (2021), eudaemnoic *wellbeing* primarily consists of the awareness of being able to act autonomously and successfully, master challenges, and find meaning in life. This definition emphasizes the relationship between wellbeing and the *SOC* components *Meaningfulness* and *Manageability*. *Hedonic wellbeing*, on the other hand, encompasses affective experiences in the sense of avoiding unpleasantness and striving for positive feelings. Clarifying the differential relationships and distinguishability of *hedonic* and *eudaimonic wellbeing* from *Neuroticism, SOC-CP*, and *SOC-MM* in relation to *Sexual satisfaction* is therefore an interesting area for further research. A distinction between *hedonic* (i.e., pleasure- and satisfaction-oriented) and *eudaemonic* (i.e., oriented toward intimate, meaningful interpersonal relationships; communion-oriented) *sexual wellbeing* (Yoo et al., 2014) would also be helpful in enhancing our understanding of the constructs *Sexual satisfaction* and health.

*Sexual satisfaction*, as measured using the self-centered subscale of the NSSS, could be modeled as a strong second-order source of variance for the sub-aspects *Intensity, Emotion* and *Orgasm* (variance explained: 79, 83, and 47%). This strengthens the model assumption that *Sexual satisfaction* can be considered a unique construct, but that it is necessary to conceptually distinguish between the corresponding sub-facets to realize the nature of the construct appropriately.

### 4.1 Limitations

Since the data were collected cross-sectionally in an ad hoc sample, limitations of internal and external validity need to be considered (Shadish et al., 2002). Although the latent regression paths in SEM are theoretically based, no empirical certainty can be gained regarding the causal directions of effects. The evidence for the mediator function of *SOC* in particular is higher when using latent path modeling than when using alternative correlation statistical analyses (Kline, 2016), but lower than when using a randomized controlled study design.

When considering the external validity or generalizability of the findings, the specific characteristics of the sample must be regarded. For example, women aged between 20 and 30, as well as people who consider themselves to be socioeconomically advantaged, are overrepresented. Note that during data collection, people of different sexual orientations were specifically targeted (e.g. via contact points for LGBTIQ+ individuals) in order to ensure a high degree of variance in the analysis characteristics. Accordingly, people with bi- and homosexual orientation are overrepresented in the study sample. This may contribute to confounding biases and limited generalizability (Wirtz, 2018). It is well documented, for example, that sexual minorities experience mental stress (e.g. depressive symptoms) more frequently. The findings reported by Brett et al. (2025) suggest that LGBTIQ+ populations experience a reduced sense of coherence compared to cisgender heterosexuals. This is in line with lower *SOC* values in our study sample (Table 7). Experiencing, anticipating and internalizing stigmatization in society results in higher psychological stress such as sexual minority stress, but also in more frequent occurrence of physical illness (Frost et al., 2015; Kasprowski et al., 2021; Merz et al., 2023). Vulnerability of LGBTIQ+ individuals can therefore be considered to be increased. *Sexual satisfaction* and sexual health could be also impaired when comparing with heteronormative individuals, not only by these mediating adverse conditions, but also explicit aggravating factors such as sexual shame and internalized homonegativity (Cienfuegos-Szalay et al., 2022). However, no evidence of structural inequality in terms of *Sexual satisfaction* was found in the present sample.

It must be assumed that selection effects are caused due to voluntary participation. Sexually inactive individuals (reduced interest in participation) or individuals who are less open about their sexual experiences, possibly due to embarrassment and shame (Waling et al., 2022), may be underrepresented, which promotes thematic structural inhomogeneity in the sample (Shadish et al., 2002). Accordingly, ten out of 232 people abandoned the survey on the page where the sexuality-related questions began. However, comparisons between sexually inactive individuals in the sample over the past month (26.9% women; 32.7% men) and the representative survey by Hoy et al. (2018) show only minor differences (42.4% women; 31.5% men), even though they have a significantly higher average age, which is associated with lower sexual activity. Common biases such as recall bias due to retrospective questioning of periods over 12 months, a tendency toward social desirability and self-serving bias cannot be ruled out either (Wirtz, 2018).

In the confirmatory analysis of the instruments used, data-driven modifications were necessary to achieve adequate model fits. Cross-validation of the findings would have been desirable but would have required a larger sample size (Kline, 2016). In future studies, the implications of the model adjustments for the construct validity of the assessment scales should be examined critically.

The economic testing of the main hypothesis regarding the complete mediation of *SOC* is a particular strength of the study. Ensuring the structural validity of the measurement models of all individual scales in advance increases the power of the subsequent nested model comparisons (Kline, 2016). In the nested model comparison, the significance of all paths that would contradict complete mediation was also tested in an integrated manner using a single significance test.

## 5 Conclusion

Sexual health is an integral facet of human bio-psycho-social health status, but it is only rarely considered in health studies. *Sexual satisfaction* represents individuals’ sexual affective experience and the subjective quality of one’s own sexuality. The NSSS is an assessment tool for measuring *Sexual satisfaction* in a systematic way. Our findings show that, according to the operationalization of the NSSS, sexual health should be understood as a construct with three the sub-facets referring to satisfaction with *Intensity*, *Emotion*, and *Orgasm*. The specified model allowed to identify evidence that the *SOC* sub-facets *Manageability and Meaningfulness* can be considered essential predictors of *Sexual satisfaction*. The significant predictive value of the personality traits *Conscientiousness*, *Agreeableness* and *Neuroticism* for *Sexual satisfaction* proved to be fully mediated by *SOC*. Accordingly, the findings suggest that *SOC* plays a key role in the path structure examined. Future research should investigate more closely whether *SOC* sub-facets (i) are directly and specifically decisive for one’s own sexual behavior and sexual experience or (ii) whether they can be considered more generically beneficial—for sexuality in the same way as for any other health-related behavior.

## Data Availability

The raw data supporting the conclusions of this article will be made available by the authors, without undue reservation.
