# Supplementary material for: Sense of coherence as moderator of the predictive power of personality variables on sexual satisfaction—a structural equation modeling approach
Source: Front Psychol. 2026 Feb 5;17:1673425. doi: 10.3389/fpsyg.2026.1673425 (PMC12916591; doi:10.3389/fpsyg.2026.1673425)
Supplement: Supplementary file 1 [file Table_1.docx]

**Supplement: Item contents**

| Label | Item |
| --- | --- |
| ***SOC-L9*** | |
| **SOC (Manageability/Meaningfulness)** (ω=0.825) | |
| SOC01 | When you think about your life. you very often…  1 = ask yourself why you exist at all  7 = feel how good it is to be alive |
| SOC02 | Doing the things you do every day is…  1 = a source of pain and boredom  7 = a source of deep pleasure and satisfaction |
| SOC03 | When you do something that gives you a good feeling…  1 = it’s certain that something will happen to spoil the feeling  7 = it’s certain that you’ll go on feeling good |
| SOC04 | You expect for the future that your own life...  1 = without any meaning or purpose  7 = full of meaning or purpose |
| SOC05 | Many people – even those with a strong character – sometimes feel like sad sacks (losers) in  certain situations. How often have you felt this way in the past…  1 = very often; 7 = very seldom or never |
| SOC06 | When you think of the difficulties you are likely to face in important aspects of your life. do you  have the feeling that…  1 = you won’t succeed in overcoming the difficulties 7 = you will always succeed in overcoming the difficulties |
| SOC07 | How often do you have the feeling that there’s little meaning in the things you do in your daily  life?  1 = very often; 7 = very seldom or never |
| **Sense of comprehensibility** (Cronbach’s α=0.650) | |
| Com01 | Do you have a feeling that you are in an unfamiliar situation and don’t know what to do?  1 = very often; 7 = very seldom or never |
| Com02 | Do you have very mixed-up feelings and ideas?  1 = very often; 7 = very seldom or never |
|  | |
| ***NSSS (McDonald’s ω=0.893)***  ***self-/centered focused subscale; 1=not at all satisfied; 5= extremely satisfied***  *Original: Thinking about your sex life during the last six months please rather your satisfaction with the follow aspects… / How satisfied are you with…* | |
| **Intensity** | |
| Int01 | The intensity of my sexual arousal. |
| Int02 | My “letting go” and surrender to sexual pleasure during sex. |
| Int03 | My focus/concentration during sexual activity. |
| Int04 | The way I sexually react to my partner. |
| Int05 | The pleasure I provide to my partner. |
| **Emotionality / Reciprocity** | |
| Em01 | My emotional opening up in sex. |
| EM02 | My mood after sexual activity. |
| **Orgasm - Satsifaction** | |
| Og04 | The quality of my orgasms. |
| Og05 | The frequency of my orgasms. |
| **Deleted** | |
| - | My body’s sexual functioning. |
